# Potassium aqua­terbium(III) oxalate sulfate

**DOI:** 10.1107/S1600536809019679

**Published:** 2009-06-06

**Authors:** Ya-Guang Sun, Mei-yan Guo, Gang Xiong, Bing Jiang, Lei Wang

**Affiliations:** a Laboratory of Coordination Chemistry, Shenyang Institute of Chemical Technology, Shenyang 110142, People’s Republic of China

## Abstract

Single crystals of KTb(C_2_O_4_)(SO_4_)(H_2_O), potassium aqua­terbium(III) oxalate sulfate, were obtained under hydro­thermal conditions. In the crystal structure, the Tb(III) atom is coordinated by four O atoms from two oxalate anions, three O atoms from three sulfate anions and one O atom from a water mol­ecule within a TbO_8_ distorted square antiprismatic coordination. The potassium and terbium(III) atoms are bridged by the oxalate and sulfate groups, forming a three-dimensional structure. The coordination mode of the oxalate  has not yet been reported. O—H⋯O hydrogen bonding between the water molecules and the oxygen atoms of oxalate and sulfate anions is also observed.

## Related literature

For oxaltes and their coordination modes, see: Audebrand *et al.* (2003 ▶); Dean *et al.* (2004 ▶); Lu *et al.* (2004 ▶).

## Experimental

### 

#### Crystal data

KTb(C_2_O_4_)(SO_4_)(H_2_O)
*M* *_r_* = 400.14
Monoclinic, 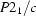

*a* = 6.5274 (13) Å
*b* = 8.5072 (17) Å
*c* = 14.591 (4) Å
β = 112.65 (3)°
*V* = 747.7 (3) Å^3^
*Z* = 4
Mo *K*α radiation
μ = 10.32 mm^−1^
*T* = 113 K
0.06 × 0.04 × 0.02 mm

#### Data collection

Bruker SMART 1000 CCD diffractometer
Absorption correction: multi-scan (*SADABS*; Sheldrick, 2003 ▶) *T*_min_ = 0.576, *T*_max_ = 0.820
4812 measured reflections
1317 independent reflections
1079 reflections with *I* > 2σ(*I*)
*R*_int_ = 0.057

#### Refinement

*R*[*F* ^2^ > 2σ(*F* ^2^)] = 0.023
*wR*(*F* ^2^) = 0.051
*S* = 1.05
1317 reflections
127 parameters
24 restraints
H-atom parameters constrained
Δρ_max_ = 0.92 e Å^−3^
Δρ_min_ = −0.78 e Å^−3^

### 

Data collection: *SMART* (Bruker, 2001 ▶); cell refinement: *SAINT* (Bruker, 2001 ▶); data reduction: *SAINT*; program(s) used to solve structure: *SHELXS97* (Sheldrick, 2008 ▶); program(s) used to refine structure: *SHELXL97* (Sheldrick, 2008 ▶); molecular graphics: *DIAMOND* (Brandenburg, 1998 ▶); software used to prepare material for publication: *SHELXTL* (Sheldrick, 2008 ▶).

## Supplementary Material

Crystal structure: contains datablocks I, global. DOI: 10.1107/S1600536809019679/br2105sup1.cif

Structure factors: contains datablocks I. DOI: 10.1107/S1600536809019679/br2105Isup2.hkl

Additional supplementary materials:  crystallographic information; 3D view; checkCIF report

## Figures and Tables

**Table 1:** Hydrogen-bond geometry (Å, °)

*D*—H⋯*A*	*D*—H	H⋯*A*	*D*⋯*A*	*D*—H⋯*A*
O9—H9*A*⋯O2^i^	0.96 (6)	1.88 (4)	2.731 (4)	145 (5)
O9—H9*B*⋯O3^ii^	0.96 (6)	1.86 (3)	2.787 (5)	160 (9)
O9—H9*B*⋯O7^i^	0.97 (6)	2.83 (9)	3.149 (5)	100 (6)

Symmetry codes: (i) 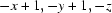; (ii) 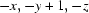.

**Fig. 1.**
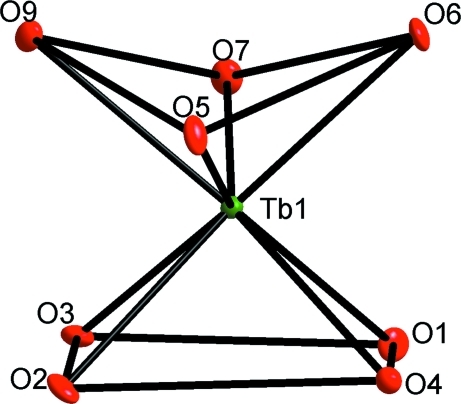
A view of the environment of (a) the Tb atom, The symmetry codes are in Table 1.Displacement ellipsoids are drawn at the 50% probability level.

**Fig. 2.**
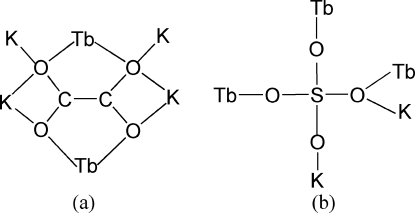
A view of coordination modes of (a)the Oxalate and (b) the sulfate anion.

**Fig. 3.**
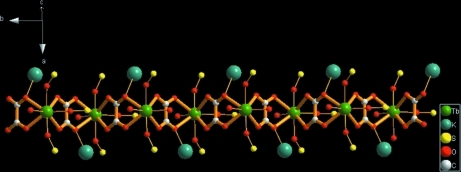
The one-dimensional chain structure of the KTb(SO4)(C2O4)(H2O) along c axis(hydrogen atoms omitted).

**Fig. 4.**
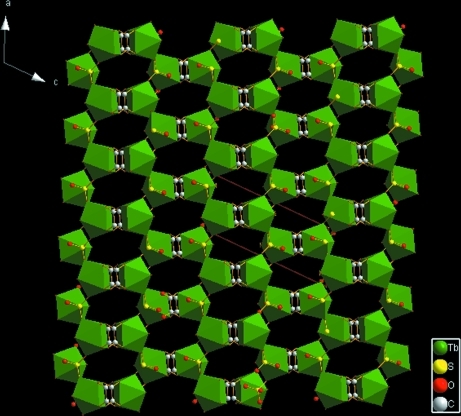
The structural arrangement of KTb(SO4)(C2O4)(H2O) along b axis(hydrogen and potassium atoms omitted).The green polyhedras are TbO8.

## supplementary crystallographic information

### Comment

Recently, rationally design of novel inorganic componds based on alkali metal ions and rare earth ions are currently of great interest because of their potential applications in photoluminescent fields. For purpose of enriching the chemistry field of this compound family, we have successfully synthesized the title compound.

In the title compound, the coordination environments of the rare earth Tb^III^ cations consist of eight O atoms which are associated with one water molecule, two sulfate groups and two oxalates. Tb^III^ cations are at the shared apex of two dicapped rectangular pyramids (Fig.1). The K^+^ cations are surrounded by nine O atoms, including one water O atom, six O atoms from oxalates and two O atoms from sulfate groups.

Oxalates are of considerable interest because many of them are natural minerals and in addition, the oxalate anion can adopt different coordination modes to bind metals to form infinite chains, sheets and networks, leading to the rich structural chemistry (Lu *et al.*, 2004; Dean *et al.*, 2004; Audebrand *et al.*, 2003). In the title compound, the oxalate ligand has an unique coordination mode (κ^3^-κ^2^-µ_4_)-(κ^3^-κ^2^-µ_4_)-µ_6_-ox^2-^. Fig.2 shows coordination mode of the oxalate and sulfate ligands in the title compuond.

Two adjacent Tb^III^ ions were connected through the oxalates to form one-dimensional chain structure (see Fig.3), and then were connected through the sulfate anions and water molecules to form the three-dimensional framework (see Fig.4).

### Experimental

A mixture of FeSO_4_.7H_2_O (0.1 mmol),Tb(NO_3_)_3_.5H_2_O (0.1 mmol) and oxalic acid (0.2 mmol) in H_2_O (10 mL) was adjust to pH=6.8 with KOH aqueous solution, sealed in a 25 mL Teflon-lined bomb at 430 K for 4 days and then slowly cooled to room temperature at a rate of 5° K per hour. Colorless block crystals were obtained by filtration.The structure was determined by single-crystal diffraction.

### Refinement

Water H atoms were located in a difference Fourier maps and refined to restraint with O—H distance of 0.97Å and Uĩso(H) = 1.2Ueq(O). In order improve the *R* and *wR* factors,weak diffraction reflections in high 2 theta angles were omitted.Because of difficulties in obtaining convergence in the refinement the anisotropy of the atomic displacement parameters of some O and C atoms were restrained.

### Crystal data

**Table:**

KTb(C_2_O_4_)(SO_4_)(H_2_O)	*F*(000) = 744
*M**_r_* = 400.14	*D*_x_ = 3.554 Mg m^−^^3^
Monoclinic, *P*2_1_/*c*	Mo *K*α radiation, λ = 0.71073 Å
Hall symbol: -P 2ybc	Cell parameters from 1317 reflections
*a* = 6.5274 (13) Å	θ = 2.8–27.2°
*b* = 8.5072 (17) Å	µ = 10.32 mm^−^^1^
*c* = 14.591 (4) Å	*T* = 113 K
β = 112.65 (3)°	Block, colorless
*V* = 747.7 (3) Å^3^	0.06 × 0.04 × 0.02 mm
*Z* = 4	

### Data collection

**Table:**

Bruker SMART 1000 CCD diffractometer	1317 independent reflections
Radiation source: rotating anode	1079 reflections with *I* > 2σ(*I*)
confocal	*R*_int_ = 0.057
ω scans	θ_max_ = 25.0°, θ_min_ = 2.8°
Absorption correction: multi-scan (*SADABS*; Sheldrick, 2003)	*h* = −7→7
*T*_min_ = 0.576, *T*_max_ = 0.820	*k* = −9→10
4812 measured reflections	*l* = −17→17

### Refinement

**Table:**

Refinement on *F*^2^	Primary atom site location: structure-invariant direct methods
Least-squares matrix: full	Secondary atom site location: difference Fourier map
*R*[*F*^2^ > 2σ(*F*^2^)] = 0.023	Hydrogen site location: inferred from neighbouring sites
*wR*(*F*^2^) = 0.051	H-atom parameters constrained
*S* = 1.05	*w* = 1/[σ^2^(*F*_o_^2^) + (0.0171*P*)^2^] where *P* = (*F*_o_^2^ + 2*F*_c_^2^)/3
1317 reflections	(Δ/σ)_max_ = 0.001
127 parameters	Δρ_max_ = 0.92 e Å^−^^3^
24 restraints	Δρ_min_ = −0.78 e Å^−^^3^

### Special details

**Table:**

Geometry. All e.s.d.'s (except the e.s.d. in the dihedral angle between two l.s. planes) are estimated using the full covariance matrix. The cell e.s.d.'s are taken into account individually in the estimation of e.s.d.'s in distances, angles and torsion angles; correlations between e.s.d.'s in cell parameters are only used when they are defined by crystal symmetry. An approximate (isotropic) treatment of cell e.s.d.'s is used for estimating e.s.d.'s involving l.s. planes.
Refinement. Refinement of *F*^2^ against ALL reflections. The weighted *R*-factor *wR* and goodness of fit *S* are based on *F*^2^, conventional *R*-factors *R* are based on *F*, with *F* set to zero for negative *F*^2^. The threshold expression of *F*^2^ > σ(*F*^2^) is used only for calculating *R*-factors(gt) *etc*. and is not relevant to the choice of reflections for refinement. *R*-factors based on *F*^2^ are statistically about twice as large as those based on *F*, and *R*- factors based on ALL data will be even larger.

### Fractional atomic coordinates and isotropic or equivalent isotropic displacement parameters (Å^2^)

**Table:**

	*x*	*y*	*z*	*U*_iso_*/*U*_eq_	
Tb1	0.33208 (4)	0.24112 (3)	0.086605 (17)	0.00425 (11)	
K1	0.9670 (2)	−0.15405 (14)	0.19899 (9)	0.0144 (3)	
S1	0.7406 (2)	0.16467 (15)	−0.02448 (9)	0.0057 (3)	
O1	0.2682 (6)	0.1255 (4)	0.2251 (3)	0.0079 (8)	
O2	0.6044 (6)	0.4446 (4)	0.1557 (2)	0.0074 (8)	
O3	0.2189 (6)	0.4466 (4)	0.1717 (2)	0.0079 (8)	
O4	0.6523 (6)	0.1083 (4)	0.1984 (3)	0.0065 (8)	
O5	0.5568 (7)	0.2364 (4)	−0.0040 (3)	0.0077 (8)	
O6	0.7540 (6)	−0.0053 (4)	−0.0015 (3)	0.0080 (8)	
O7	0.9493 (7)	0.2388 (4)	0.0409 (3)	0.0096 (9)	
O8	0.7035 (7)	0.1876 (5)	−0.1285 (3)	0.0119 (9)	
O9	0.1774 (6)	0.4070 (4)	−0.0544 (3)	0.0072 (8)	
H9A	0.285 (8)	0.475 (6)	−0.063 (5)	0.04 (2)*	
H9B	0.047 (6)	0.448 (9)	−0.107 (5)	0.13 (4)*	
C1	0.4137 (9)	0.0364 (6)	0.2801 (4)	0.0043 (11)	
C2	0.3644 (9)	0.5312 (6)	0.2329 (4)	0.0052 (11)	

### Atomic displacement parameters (Å^2^)

**Table:**

	*U*^11^	*U*^22^	*U*^33^	*U*^12^	*U*^13^	*U*^23^
Tb1	0.00438 (17)	0.00457 (17)	0.00411 (16)	0.00031 (11)	0.00197 (12)	0.00024 (10)
K1	0.0141 (8)	0.0162 (7)	0.0164 (7)	−0.0038 (6)	0.0099 (6)	−0.0061 (5)
S1	0.0054 (8)	0.0063 (7)	0.0061 (7)	−0.0002 (6)	0.0029 (6)	−0.0010 (5)
O1	0.006 (2)	0.009 (2)	0.010 (2)	0.0033 (17)	0.0045 (18)	0.0001 (15)
O2	0.010 (2)	0.0070 (19)	0.008 (2)	−0.0028 (17)	0.0067 (18)	−0.0041 (16)
O3	0.008 (2)	0.009 (2)	0.006 (2)	−0.0019 (17)	0.0013 (18)	−0.0020 (15)
O4	0.0068 (12)	0.0062 (11)	0.0066 (11)	−0.0001 (9)	0.0028 (9)	−0.0002 (8)
O5	0.011 (2)	0.0047 (19)	0.013 (2)	0.0011 (16)	0.0096 (18)	−0.0002 (14)
O6	0.011 (2)	0.0035 (19)	0.010 (2)	−0.0010 (17)	0.0048 (17)	−0.0014 (14)
O7	0.008 (2)	0.009 (2)	0.012 (2)	0.0002 (17)	0.0041 (18)	0.0008 (16)
O8	0.016 (3)	0.014 (2)	0.008 (2)	−0.0015 (19)	0.0071 (19)	0.0010 (16)
O9	0.0063 (12)	0.0077 (12)	0.0083 (11)	−0.0003 (9)	0.0036 (9)	0.0017 (8)
C1	0.0048 (14)	0.0036 (14)	0.0043 (13)	0.0005 (9)	0.0015 (10)	−0.0011 (9)
C2	0.0052 (14)	0.0046 (14)	0.0057 (14)	0.0003 (9)	0.0019 (10)	0.0010 (9)

### Geometric parameters (Å, °)

**Table:**

Tb1—O6^i^	2.311 (4)	S1—O5	1.476 (3)
Tb1—O5	2.323 (3)	S1—O6	1.480 (4)
Tb1—O7^ii^	2.325 (4)	O1—C1	1.238 (6)
Tb1—O9	2.375 (4)	O1—K1^vii^	2.900 (3)
Tb1—O4	2.382 (4)	O1—K1^ii^	3.016 (4)
Tb1—O2	2.406 (4)	O2—C1^vii^	1.261 (5)
Tb1—O3	2.419 (3)	O2—K1^viii^	2.909 (4)
Tb1—O1	2.423 (3)	O3—C2	1.250 (6)
K1—O8^iii^	2.733 (4)	O3—K1^vii^	2.744 (3)
K1—O3^iv^	2.744 (3)	O4—C2^iv^	1.238 (6)
K1—O1^iv^	2.900 (3)	O4—K1^viii^	3.105 (4)
K1—O9^i^	2.903 (4)	O6—Tb1^i^	2.311 (4)
K1—O2^v^	2.909 (4)	O7—Tb1^vi^	2.325 (4)
K1—O6	2.992 (4)	O8—K1^iii^	2.733 (4)
K1—O1^vi^	3.016 (4)	O9—K1^i^	2.903 (4)
K1—O4	3.032 (4)	O9—H9A	0.96 (6)
K1—O4^v^	3.105 (4)	O9—H9B	0.97 (6)
K1—C2^iv^	3.132 (5)	C1—O2^iv^	1.261 (5)
K1—C1^vi^	3.141 (6)	C1—C2^iv^	1.532 (7)
K1—S1^iii^	3.7251 (18)	C2—O4^vii^	1.238 (6)
S1—O8	1.455 (4)	C2—C1^vii^	1.532 (7)
S1—O7	1.470 (4)		
			
O6^i^—Tb1—O5	75.84 (12)	O1^vi^—K1—O4	80.09 (10)
O6^i^—Tb1—O7^ii^	80.00 (13)	O8^iii^—K1—O4^v^	60.90 (11)
O5—Tb1—O7^ii^	132.93 (13)	O3^iv^—K1—O4^v^	110.83 (11)
O6^i^—Tb1—O9	96.65 (12)	O1^iv^—K1—O4^v^	80.67 (10)
O5—Tb1—O9	70.62 (12)	O9^i^—K1—O4^v^	80.98 (10)
O7^ii^—Tb1—O9	72.86 (12)	O2^v^—K1—O4^v^	57.98 (10)
O5—Tb1—O4	78.58 (12)	O6—K1—O4^v^	136.94 (9)
O7^ii^—Tb1—O4	138.98 (12)	O1^vi^—K1—O4^v^	95.21 (11)
O9—Tb1—O4	147.42 (11)	O4—K1—O4^v^	153.72 (5)
O6^i^—Tb1—O2	146.78 (11)	O8—S1—O7	111.1 (2)
O5—Tb1—O2	74.00 (11)	O8—S1—O5	109.5 (2)
O7^ii^—Tb1—O2	131.70 (12)	O7—S1—O5	108.3 (2)
O9—Tb1—O2	86.26 (13)	O8—S1—O6	109.8 (2)
O4—Tb1—O2	75.09 (13)	O7—S1—O6	108.2 (2)
O6^i^—Tb1—O3	147.54 (12)	O5—S1—O6	109.9 (2)
O5—Tb1—O3	133.59 (11)	C1—O1—Tb1	117.0 (3)
O7^ii^—Tb1—O3	69.30 (12)	C1—O1—K1^vii^	122.4 (3)
O4—Tb1—O3	110.60 (12)	Tb1—O1—K1^vii^	110.02 (12)
O2—Tb1—O3	65.64 (12)	C1—O1—K1^ii^	84.2 (3)
O6^i^—Tb1—O1	90.64 (12)	Tb1—O1—K1^ii^	121.87 (14)
O5—Tb1—O1	144.76 (13)	K1^vii^—O1—K1^ii^	98.21 (10)
O9—Tb1—O1	144.20 (12)	C1^vii^—O2—Tb1	119.4 (3)
O4—Tb1—O1	67.90 (12)	C1^vii^—O2—K1^viii^	88.6 (3)
O2—Tb1—O1	106.20 (11)	Tb1—O2—K1^viii^	116.49 (13)
O3—Tb1—O1	71.38 (11)	C2—O3—Tb1	119.0 (3)
O8^iii^—K1—O3^iv^	155.77 (13)	C2—O3—K1^vii^	96.0 (3)
O8^iii^—K1—O1^iv^	133.26 (11)	Tb1—O3—K1^vii^	115.52 (14)
O3^iv^—K1—O1^iv^	59.99 (10)	C2^iv^—O4—Tb1	118.6 (3)
O8^iii^—K1—O9^i^	74.43 (11)	C2^iv^—O4—K1	83.0 (3)
O3^iv^—K1—O9^i^	128.67 (11)	Tb1—O4—K1	139.97 (14)
O1^iv^—K1—O9^i^	74.15 (10)	C2^iv^—O4—K1^viii^	105.0 (3)
O8^iii^—K1—O2^v^	68.22 (11)	Tb1—O4—K1^viii^	110.44 (12)
O3^iv^—K1—O2^v^	87.98 (11)	K1—O4—K1^viii^	93.58 (10)
O1^iv^—K1—O2^v^	114.22 (11)	S1—O5—Tb1	149.7 (2)
O9^i^—K1—O2^v^	134.04 (11)	S1—O6—Tb1^i^	138.2 (2)
O8^iii^—K1—O6	79.21 (11)	S1—O6—K1	126.8 (2)
O3^iv^—K1—O6	112.22 (11)	Tb1^i^—O6—K1	94.79 (12)
O1^iv^—K1—O6	122.12 (12)	S1—O7—Tb1^vi^	144.5 (2)
O9^i^—K1—O6	72.82 (10)	S1—O8—K1^iii^	122.7 (2)
O2^v^—K1—O6	123.02 (10)	Tb1—O9—K1^i^	95.73 (12)
O8^iii^—K1—O1^vi^	63.92 (11)	Tb1—O9—H9A	113 (4)
O3^iv^—K1—O1^vi^	96.12 (11)	K1^i^—O9—H9A	113 (4)
O1^iv^—K1—O1^vi^	151.31 (6)	Tb1—O9—H9B	149 (4)
O9^i^—K1—O1^vi^	133.59 (10)	K1^i^—O9—H9B	75 (6)
O2^v^—K1—O1^vi^	44.18 (10)	H9A—O9—H9B	97.7 (13)
O6—K1—O1^vi^	79.92 (11)	O1—C1—O2^iv^	126.4 (5)
O8^iii^—K1—O4	134.96 (11)	O1—C1—C2^iv^	117.6 (4)
O3^iv^—K1—O4	45.03 (10)	O2^iv^—C1—C2^iv^	116.0 (5)
O1^iv^—K1—O4	91.10 (10)	O4^vii^—C2—O3	127.1 (5)
O9^i^—K1—O4	120.90 (11)	O4^vii^—C2—C1^vii^	117.8 (5)
O2^v^—K1—O4	104.49 (11)	O3—C2—C1^vii^	115.0 (4)
O6—K1—O4	68.10 (9)		

Symmetry codes: (i) −*x*+1, −*y*, −*z*; (ii) *x*−1, *y*, *z*; (iii) −*x*+2, −*y*, −*z*; (iv) −*x*+1, *y*−1/2, −*z*+1/2; (v) −*x*+2, *y*−1/2, −*z*+1/2; (vi) *x*+1, *y*, *z*; (vii) −*x*+1, *y*+1/2, −*z*+1/2; (viii) −*x*+2, *y*+1/2, −*z*+1/2.

### Hydrogen-bond geometry (Å, °)

**Table:**

*D*—H···*A*	*D*—H	H···*A*	*D*···*A*	*D*—H···*A*
O9—H9A···O2^ix^	0.96 (6)	1.88 (4)	2.731 (4)	145 (5)
O9—H9B···O3^x^	0.96 (6)	1.86 (3)	2.787 (5)	160 (9)
O9—H9B···O7^ix^	0.97 (6)	2.83 (9)	3.149 (5)	100 (6)

Symmetry codes: (ix) −*x*+1, −*y*+1, −*z*; (x) −*x*, −*y*+1, −*z*.

## References

[bb1] Audebrand, N., Raite, S. & Louer, D. (2003). *Solid State Sci* **5**, 783–794.

[bb2] Brandenburg, K. (1998). *DIAMOND* Crystal Impact GbR, Bonn, Germany.

[bb3] Bruker (2001). *SAINT* and *SMART* Bruker AXS Inc., Madison, Wisconsin, USA.

[bb4] Dean, P. A. W., Craig, D., Dance, I., Russell, V. & Scudder, M. (2004). *Inorg. Chem* **43**, 443–449. 10.1021/ic034988k 14731006

[bb5] Lu, J., Li, Y., Zhao, K., Xu, J.-Q., Yu, J.-H., Li, G.-H., Zhang, X., Bie, H.-Y. & Wang, T.-G. (2004). *Inorg. Chem. Commun.* **7**, 1154–1156.

[bb6] Sheldrick, G. M. (2003). *SADABS* University of Göttingen, Germany.

[bb7] Sheldrick, G. M. (2008). *Acta Cryst.* A**64**, 112–122. 10.1107/S0108767307043930 18156677

